# Identification and Analysis of the GMC Oxidoreductase Family Genes in *Cnaphalocrocis medinalis* and Their Response to Spinetoram

**DOI:** 10.3390/insects16121272

**Published:** 2025-12-15

**Authors:** Conghui Xiao, Pengqi Quan, Jian Zhu, Haixia Lei, Kailong Li, Xin Zhao, Daohong Zhu, Yang Zeng, Mingyong Ma

**Affiliations:** 1 College of Life Science and Technology, Central South University of Forestry and Technology, Changsha 410004, China; 18390643006@163.com (C.X.); dhzhu@csuft.edu.cn (D.Z.); 2 Institute of Plant Protection, Hunan Academy of Agriculture Sciences, Changsha 410125, China; 13213502940@163.com (P.Q.); zjnjau@163.com (J.Z.); lannuolkl@foxmail.com (K.L.); 3 Xinyang Academy of Agricultural Sciences, Xinyang 464000, China; lhx2014102085@163.com; 4 Horticulture Research Institute of Tonghua City, Tonghua 134001, China; zxmt107@163.com

**Keywords:** *Cnaphalocrocis medinalis*, GMC oxidoreductase, gene family, spinetoram, insecticide response

## Abstract

The glucose–methanol–choline (GMC) oxidoreductase superfamily constitutes a crucial group of enzymes involved in diverse physiological processes in insects. However, a systematic investigation of this gene family in the rice leaf roller, *Cnaphalocrocis medinalis*—a major migratory rice pest—remains lacking. This study identified 54 CmGMC genes in *C. medinalis*. Genomic analysis revealed its uneven chromosomal distribution, with a conserved 12-gene tandem cluster on chromosome 23. Phylogenetic analysis classified the CmGMC genes into distinct clades, clarifying their evolutionary relationships with GMC homologs in other species. Furthermore, spatiotemporal expression profiling revealed expression of 36 CmGMC genes across all developmental stages and tissues examined, indicating the high transcriptional activity of GMC oxidoreductase genes in *C. medinalis*. To investigate their role in insecticide response, we examined changes in CmGMC expression following spinetoram treatment. At 48 h post treatment, 20 and 8 genes were significantly upregulated and downregulated, respectively, indicating that specific GMC oxidoreductases may play crucial roles in the molecular response of *C. medinalis* to spinetoram. This study provides a foundation for understanding the biological functions of GMC oxidoreductases in *C. medinalis* and reveals their response to the insecticide spinetoram.

## Simple Summary

Rice leaf folder, *Cnaphalocrocis medinalis*, is a devastating rice crop pest, and its evolving resistance to insecticides poses a substantial challenge to control efforts. The glucose–methanol–choline (GMC) oxidoreductase superfamily comprises important enzymes involved in various physiological processes in insects, but their specific roles in *C. medinalis* remain largely unknown. This study presents the first systematic identification of 54 GMC genes in this species, revealing an uneven chromosomal distribution of these genes, including a conserved tandem cluster of 12 genes. Expression profiling revealed most GMC genes to be transcriptionally active, and exposure to the insecticide spinetoram significantly altered the expression of several genes, with 20 upregulated and 8 downregulated. This suggests that specific GMC oxidoreductases may play key roles in the molecular response of *C. medinalis* to insecticide. Our findings provide valuable genetic resources and insights for exploring novel pest management strategies.

## 1. Introduction

Redox reactions are crucial biochemical reactions in living organisms, with vital roles in sustaining biological processes such as metabolism [1] and respiration [2]. These processes also require catalysis by oxidoreductases. Based on analysis of sequences of glucose dehydrogenase from *Drosophila melanogaster*, choline dehydrogenase from *Escherichia coli*, glucose oxidase from *Aspergillus niger*, and methanol oxidase from *Hansenula polymorpha*, Cavener et al. designated enzymes featuring an N-terminal FAD-binding βαβ-fold domain composed of 30 amino acids as the glucose–methanol–choline (GMC) oxidoreductase superfamily [3]. Although the maximum sequence similarity among these enzymes is only 32%, and their diverse catalytic substrates range from various sugars and alcohols to cholesterol and choline, the overall reaction mechanisms of these oxidoreductases are still conserved [4,5,6].

The earliest functional studies of the GMC family primarily focused on lower organisms, such as *Penicillium amagasakiense* and *Aspergillus niger* [7]. More recently, an increasing number of GMC oxidoreductase genes have been identified in insects, the first being the glucose dehydrogenase gene in *D. melanogaster* [3]. More recently published research on GMC family gene functions has mainly focused on glucose oxidase, NinaG, and ecdysone oxidase, among family members. Glucose dehydrogenases participate in glucose metabolism, catalyzing the conversion of glucose to glucono-d-lactone [8]. They also play important roles in egg development in *Aedes aegypti* [9], reproduction in the brown planthopper (*Laodelphax striatellus*) [10] and *Drosophila* [11], lifespan in *Anopheles stephensi* [12] and *Drosophila* [13], and immunity in *Manduca sexta* [14,15]. Glucose oxidase has also been implicated in insect feeding [16,17] and insect–plant interactions [18,19]. The NinaG oxidoreductase gene (belong to GMC family) is required for the synthesis of visual pigments in insect visual systems [20,21,22]. Ecdysone regulates developmental transitions in insects, and ecdysone oxidase is a key enzyme that modulates ecdysone titers [23]. Ecdysone oxidase PxEO in the diamondback moth, *Plutella xylostella*, is associated with Resistance to the bacterially derived Bt Cry1Ac toxin [24]. Ecdysone oxidase in *Drosophila* is linked to cell death through autophagy [25] and the response to malnutrition [26]. Additionally, expression of ecdysone oxidase in silk moth (*Bombyx mori*) oocytes influences the egg hatch rate of their offspring [27].

With the continuous advancement of genome sequencing technology, the identification of GMC family members has become increasingly more comprehensive. Genomic analyses of *D. melanogaster*, *A. gambiae*, *Apis mellifera*, and *Tribolium castaneum* have revealed varying numbers of GMC oxidoreductase family members (15, 15, 18, and 23, respectively). Most of these genes are arrayed in tandem within the introns of the *flotillin-2* gene. This tandem gene cluster exhibits high conservation across diverse insect taxa, suggesting critically important functions for GMC family genes in insects. In *D. melanogaster* GMC family, 12 GMC genes are located in the tandem gene cluster [28]. In the silkworm genome, 43 GMC oxidoreductase genes have been identified, most of which were the apparent product of an expansion of the GMCβ subfamily, which is largely associated with immune function [29]. Similarly, 33 GMC genes were identified in the genome of the monarch butterfly (*Danaus plexippus*), another lepidopteran. Phylogenetic analysis of the GMC family in the lepidopteran species *M. sexta*, *Pieris rapae*, *Chloridea virescens*, *Spodoptera litura*, and *D. plexippus* has also revealed expansions within the GMCβ subfamily [30].

The rice leaf folder, *C. medinalis*, is a major migratory lepidopteran pest of rice. Its larvae roll and feed on leaf tissue, reducing photosynthesis and thus causing rice yield losses [31]. As a consequence of the extensive application of chemical insecticides to control such pests, the evolution of resistance poses a major challenge to sustainable agriculture [32,33]. Between 2019 and 2022, *C. medinalis* developed low to moderate resistance to abamectin, emamectin benzoate, and spinetoram and high resistance to chlorantraniliprole, with resistance ratios reaching 64.9–113.7 [34]. Consequently, Bt transgenic insect-resistant rice has garnered substantial attention [35,36]. Simultaneously, detoxification enzyme genes, such as cytochrome P450 monooxygenases (P450s) [37,38], glutathione *S*-transferases (GSTs) [39,40], and carboxylesterases (COEs) [41], have become very active research subjects [42].

Spinetoram is a bio-insecticide derived from natural products, which primarily acts on the nicotinic acetylcholine receptors (nAChRs) and γ-aminobutyric acid (GABA) receptors in insects, disrupting nervous system function through stomach and contact toxicity [43,44]. It is highly effective against lepidopteran pests and has been widely used for both field control and laboratory studies of *C. medinalis* [45,46]. However, the molecular mechanisms by which *C. medinalis* responds to spinetoram stress through its metabolic enzyme systems remain unclear. Therefore, this insecticide was selected in this study to investigate the potential role of GMC family genes in the detoxification process. The publication of the *C. medinalis* transcriptome [47] and genome [48] has enabled the identification and analysis of gene families in this important crop pest [49,50,51]. Utilizing these genomic data, the present study identified 54 GMC oxidoreductase genes in *C. medinalis* through various methods of analysis, including conserved domain analysis, chromosomal localization, and phylogenetic analysis. Spatiotemporal expression profiles of the GMC oxidoreductase gene family were also constructed. To investigate the potential role of GMC family genes in detoxification, we measured their expression levels following spinetoram treatment, revealing that 15 GMC oxidoreductase genes were upregulated and 3 were downregulated, which suggests these genes may play important roles in the response to spinetoram observed in *C. medinalis*.

## 2. Materials and Methods

### 2.1. Data Acquisition

Genomic data, protein data, and GFF files for the following 14 insect species were downloaded from the Insectbase website (http://v2.insect-genome.com/Organism/192 (accessed on 8 December 2024)). *C. medinalis*, *B. mori*, *Ostrinia furnacalis*, *Monomorium pharaonis*, *Acromyrmex charruanus*, *Tenebrio molitor*, *Anoplophora glabripennis*, *Gryllus bimaculatus*, *Locusta migratoria*, *Eucriotettix oculatus*, *Bemisia tabaci*, *Nilaparvata lugens*, *Apolygus lucorum*, and *Ceratitis capitata*. Protein databases for four species (*D. melanogaster*, *A. gambiae*, *A. mellifera*, *T. castaneum*) and GMC sequences for six species (*Homo sapiens*, *Caenorhabditis elegans*, *A. niger*, *Penicillium amagasakiense*, *A. oryzae*, *E. coli*) were also downloaded from the previous research [28] (https://doi.org/10.1186/1471-2148-7-75 (accessed on 11 December 2024)). The Hidden Markov Model (HMM) profile (Pfam ID PF00732) used for identifying the GMC oxidoreductase family was obtained from the Pfam database (http://pfam.xfam.org/ (accessed on 11 December 2024)).

### 2.2. Identification and Nomenclature of GMC Oxidoreductase Genes

The protein annotation file of *C. medinalis* was screened using the HMM profile of the GMC family (PF00372) with TBtools (TBtools-II, Toolbox for Biologists, v2.086, Guangzhou, China) [52] at an E-value cutoff of 1E-5. Known GMC gene family sequences from *D. melanogaster* were used as query sequences for BLAST (TBtools v2.086) homology search against the *C. medinalis* protein database. The resulting sequences obtained via both methods were combined and redundant sequences were removed. Further filtering was performed using the Pfam website (https://www.ebi.ac.uk/interpro/entry/pfam/#table (accessed on 10 December 2024)). Finally, the conserved domains of the putative gene family members were verified using the NCBI CDD Search online tool (https://www.ncbi.nlm.nih.gov/Structure/bwrpsb/bwrpsb.cgi (accessed on 25 December 2024)). Sequences lacking the conserved GMC domain were identified as false positives and manually removed using TBtools. Annotation was further curated using IGV-GSAman software (v0.9.53), after which the final number of GMC genes in *C. medinalis* was ultimately determined. The identified genes were named sequentially according to their positional order along the chromosomes, arranged from the shortest to the longest chromosome. GMC genes in other species were identified according to the same methodology.

### 2.3. Analysis of Basic Information and Physicochemical Properties of the C. medinalis GMC Gene Family

Positional information on all GMC genes was obtained from the *C. medinalis* genome annotation file. Basic information, including chromosomal location, gene transcription start and stop sites, length, and strand orientation, was extracted using the GXF Gene Position & Info Extract function in TBtools. A series of amino acid physicochemical properties for each gene, including the number of amino acids, molecular weight, isoelectric point (pI), instability index, aliphatic index, and grand average of hydropathicity (GRAVY), were determined from the nucleotide sequences using the Protein Parameter Calc program.

### 2.4. Chromosomal Localization, Motif, and Conserved Domain Analysis of the C. medinalis GMC Gene Family

The chromosomal positions of all GMC genes were obtained from the *C. medinalis* genome annotation file and visualized using the Gene Location Visualize from GTF/GFF tool in TBtools. Motif analysis of the *C. medinalis* GMC gene family was performed using the MEME web tool (https://meme-suite.org/meme/ (accessed on 5 March 2025)) in conjunction with TBtools for visualization. Conserved domains were identified using the NCBI CDD database and Pfam and visualized using TBtools.

### 2.5. Phylogenetic Analysis of the C. medinalis GMC Oxidoreductase Family

A total of 134 GMC amino acid sequences were subjected to phylogenetic analysis. This set included sequences from six outgroup species (*H. sapiens*, *C. elegans*, *A. niger*, *P. amagasakiense*, *A. oryzae*, *E. coli*) and four model non-lepidopteran insects (*D. melanogaster*, *A. gambiae*, *A. mellifera*, *T. castaneum*), along with the sequences from *C. medinalis*. All sequences were aligned using the CLUSTALW method implemented in MEGA12 (v12.1.1) [53]. A phylogenetic tree was constructed with MEGA12 as well as using the maximum likelihood method with the LG + G + I model, with 1000 bootstrap replicates [54,55,56,57]. The resulting tree was subsequently visualized using the ITOL web tool (https://itol.embl.de/ (accessed on 11 September 2025)).

### 2.6. Spatiotemporal Expression of CmGMC Oxidoreductase Family Genes

Approximately 200 eggs, 10 first-instar larvae, six second-instar larvae, three third-instar larvae, three fourth-instar larvae, three fifth-instar larvae, three prepupae, three pupae, three female adults, and three male adults were collected into 2 mL cryotubes. Third-instar larvae were dissected to collect specific samples of the head, epidermis, body fluid, sericterium, gut and Malpighian tubule into 2 mL cryotubes. All 48 samples were flash-frozen in liquid nitrogen and stored at −80 °C until their later analysis. Each treatment included three biological replicates. RNA was extracted using the TRIzol method (TaKaRa, Dalian, China), and cDNA was synthesized following the instructions of the PrimeScript™ RT Reagent Kit with gDNA Eraser (Perfect Real Time) (TaKaRa). Primers for GMC family genes, designed from the *C. medinalis* genome sequence using Primer5 software (v5.0), and reference genes (*β-actin* and *RPs* 15 [47]) are listed in Table A1. PCR amplification was performed using Novozan 2× Rapid Taq Master Mix (Vazyme, Nanjing, China). Each 25 μL reaction contained 12.5 μL of Master Mix, 2 μL of cDNA template (100 ng/μL), 1 μL each of forward and reverse primer (10 μM), and 8.5 μL of sterilized water. Amplification was conducted with a thermal cycling (LTC-PCR-196; LABGIC, Puchong, Malaysai) according to the following program: 94 °C for 2 min; 30 cycles of 94 °C for 30 s, 60 °C for 30 s, 72 °C for 1 min; and a final extension at 72 °C for 10 min with the reaction held at 4 °C thereafter. After amplification, the PCR products were checked by agarose gel electrophoresis and imaged using a gel documentation system (QuickGel 6200, Monad, Wuhan, China).

### 2.7. Insecticide Treatment

A laboratory-reared population of *C. medinalis* (This population was donated by the laboratory of Professor Liu Xiangdong at Nanjing Agricultural University. It was collected in 2010 from rice fields in Nanjing, China, and has been maintained in the laboratory to date. The rearing conditions were carried out according to the method by Zhu et al. [58]) was treated with insecticide. Third-instar larvae were treated using the leaf-dipping method. Wheat leaves (15–20 cm long) were cut from plants and immersed in 0.06 mg/mL (LC_25_) and 0.12 mg/mL (LC_50_) (Table A2) solutions of spinetoram (Dow AgroSciences, Nantong, China) for 30 s and then air-dried. For the control (CK) treatment, wheat leaves were instead immersed in water. A total of 270 third-instar larvae were collected for treatment. Thirty larvae were placed on each insecticide-treated or control wheat leaf. Each treatment included three replicates. Larval survival rates were recorded at 2, 4, and 6 d post treatment. Survival was determined based on the ability of an individual larva to turn itself over when placed on its back with a small brush. Pupation rate and eclosion rate were also recorded.

### 2.8. Measurement of Relative Expression Levels of CmGMC Oxidoreductase Family Genes

Third-instar larvae were treated with spinetoram (Dow AgroSciences, Nantong, China) using the leaf-dipping method described above. One hundred and twenty third-instar larvae were collected; thirty larvae were placed on each insecticide-treated or control wheat leaf. Surviving larvae were collected into 2 mL grinding tubes at 3, 24, and 48 h post treatment, flash-frozen in liquid nitrogen, and stored at −80 °C for later use. Each treatment included three biological replicates (tubes), with each tube containing three larvae. RNA extraction was performed using the RNAiso Plus kit (TaKaRa), and cDNA synthesis was conducted using the PrimeScript™ FAST RT reagent Kit with gDNA Eraser (TaKaRa) according to the manufacturer’s instructions. The relative expression levels of GMC family genes were determined using the TB GREEN Premix Ex Taq Kit (TaKaRa) according to the manufacturer’s instructions. Quantitative real-time PCR (qRT-PCR) was performed using an Applied Biosystems by Thermo Fisher Scientific QuantStudio™ 1 Real-Time PCR Instrument (96-Well 0.2 mL Block; Thermo Fisher Scientific, Waltham, MA, USA). Primers for GMC family genes are listed in Table A1. The relative expression levels were calculated using the 2^(−ΔΔCt)^ method [59]. Each sample was run with three technical replicates.

### 2.9. Data Analysis

Data are presented as mean ± standard error (SE). Survival rate, pupation rate, eclosion rate, and gene expression data were analyzed using one-way analysis of variance (ANOVA), followed by Tukey’s test. Before ANOVA, the Shapiro–Wilk test and Levene’s test were used to confirm the normality of data distribution and the homogeneity of variances, respectively. Gene expression data that did not meet the normality assumption were square-root transformed. The significance level was set at α = 0.05 and α = 0.01. All statistical analyses were performed using DPS18.10 software [60].

## 3. Results

### 3.1. Identification of the GMC Gene Family in Cnaphalocrocis medinalis

**Figure 1:**
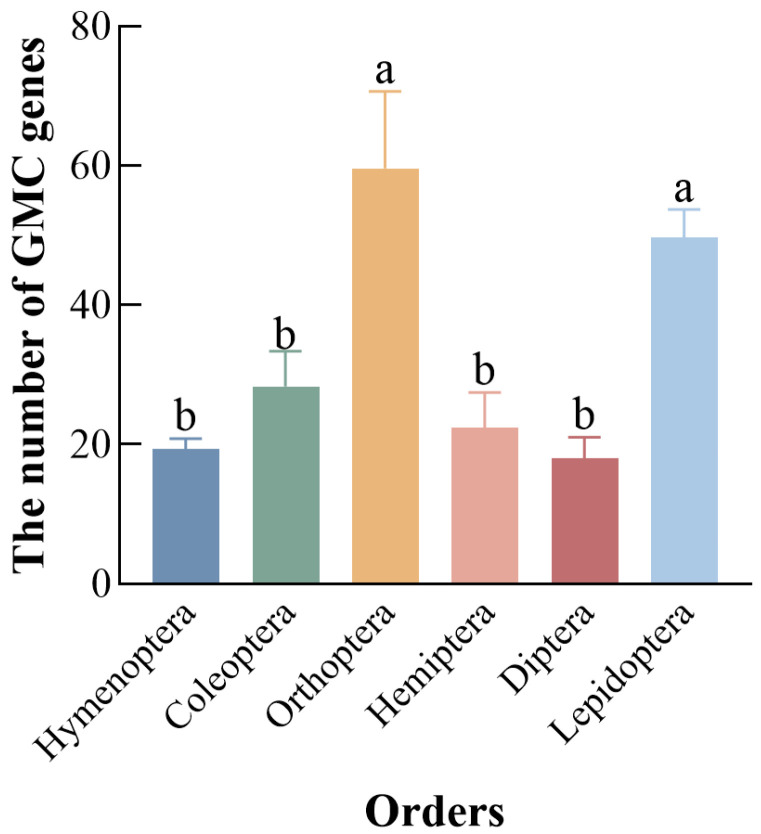
Number of GMC oxidoreductase genes across six insect orders. Different letters above bars indicate significant differences between groups according to Tukey’s test (*p* < 0.05). Data represent mean ± SE of *n* = 3.

Screening using HMMs and local BLAST identified 50 GMC genes in the *C. medinalis*. The 50 initially identified GMC genes in *C. medinalis* were assigned names *CmGMC1* to *CmGMC50* based on their chromosomal locations and positions. Conserved domain analysis revealed that *CmGMC13* lacked the characteristic GMC family conserved domain, indicating it does not actually belong to this family, despite its sequence similarity; consequently, it was excluded from subsequent analyses. Furthermore, we suspected potential mis-annotation related to over-splicing for *CmGMC17* and *CmGMC27*, as they contained multiple conserved domains. Using transcriptome data for correction with IGV-GSAman (v0.9.53), *CmGMC17* was split into four genes (*CmGMC17-1*, *CmGMC17-2*, *CmGMC17-3*, *CmGMC17-4*), and *CmGMC27* was split into three genes (*CmGMC27-1*, *CmGMC27-2*, *CmGMC27-3*), resulting in complete C- and N-terminal subsequences for the split genes. Thus, the final number of GMC gene family members in *C. medinalis* was determined to be 54 (Table A3). Among hymenopterans, 18, 21, and 19 GMC genes were identified in *A. mellifera*, *M. pharaonis*, and *A. charruanus*, respectively. There are 23, 29, 33 GMC genes in *T. castaneum*, *T. molitor*, *A. glabripennis* separately among Coleoptera, while in Orthoptera, 49, 59, and 71 genes were identified in *G. bimaculatus*, *L. migratoria*, and *E. oculatus*, respectively. Additionally, in Hemiptera, 28, 21, and 28 genes were found in *B. tabaci*, *N. lugens*, and *A. lucorum*, respectively, while among dipterans, 21, 15, and 18 genes were identified in *C. capitata*, *D. melanogaster*, and *A. gambiae*, respectively. In the lepidopteran species *B. mori* and *O. furnacalis*, 49 and 46 GMC family genes were identified, respectively (Figure 1). A comparison of the number of GMC homologs among six common insect orders revealed their greater abundance in Orthoptera (60), Lepidoptera (49), and lower abundance in Coleoptera (28), Hymenoptera (19), Hemiptera (25), and Diptera (18).

### 3.2. Physicochemical Properties of the C. medinalis GMC Gene Family

Analyzing the physicochemical properties of proteins is crucial for understanding their functions and potential applications that take advantage of these functions, e.g., in pest control; the physicochemical properties of the *C. medinalis* GMC gene family were thus analyzed using TBtools. The results indicated that the amino acid sequence lengths of the 54 CmGMC proteins ranged from 124 (*CmGMC40*) to 1036 (*CmGMC10*), with molecular weights of 13,947.8 Da (*CmGMC40*) to 127,085.0 Da (*CmGMC10*) and isoelectric points (pI) of 4.62 (*CmGMC14*) to 9.67 (*CmGMC33*) (Table 1). Among homologs, 25 proteins had a pI greater than 7, classifying them as basic proteins, while the remaining 29 had a pI less than 7, classifying them as acidic proteins. The instability index values ranged from 10.0 (*CmGMC44*) to 50.3 (*CmGMC39*). Fourteen CmGMC proteins had an instability index value exceeding 40, indicating relatively poor stability, while the remaining 40 proteins had an instability index value less than 40, indicating their relatively stable structural properties. The aliphatic index overall ranged from 61.3 (*CmGMC44*) to 105.1 (*CmGMC21*), with 11 CmGMC proteins having an aliphatic index value greater than 90, indicating their high thermal stability. The grand average of hydropathicity (GRAVY) was less than 0 for all 54 CmGMC proteins, classifying all of them as hydrophilic proteins.

### 3.3. Chromosomal Localization, Motif, and Conserved Domain Analysis of GMC Genes in C. medinalis 

**Figure 2:**
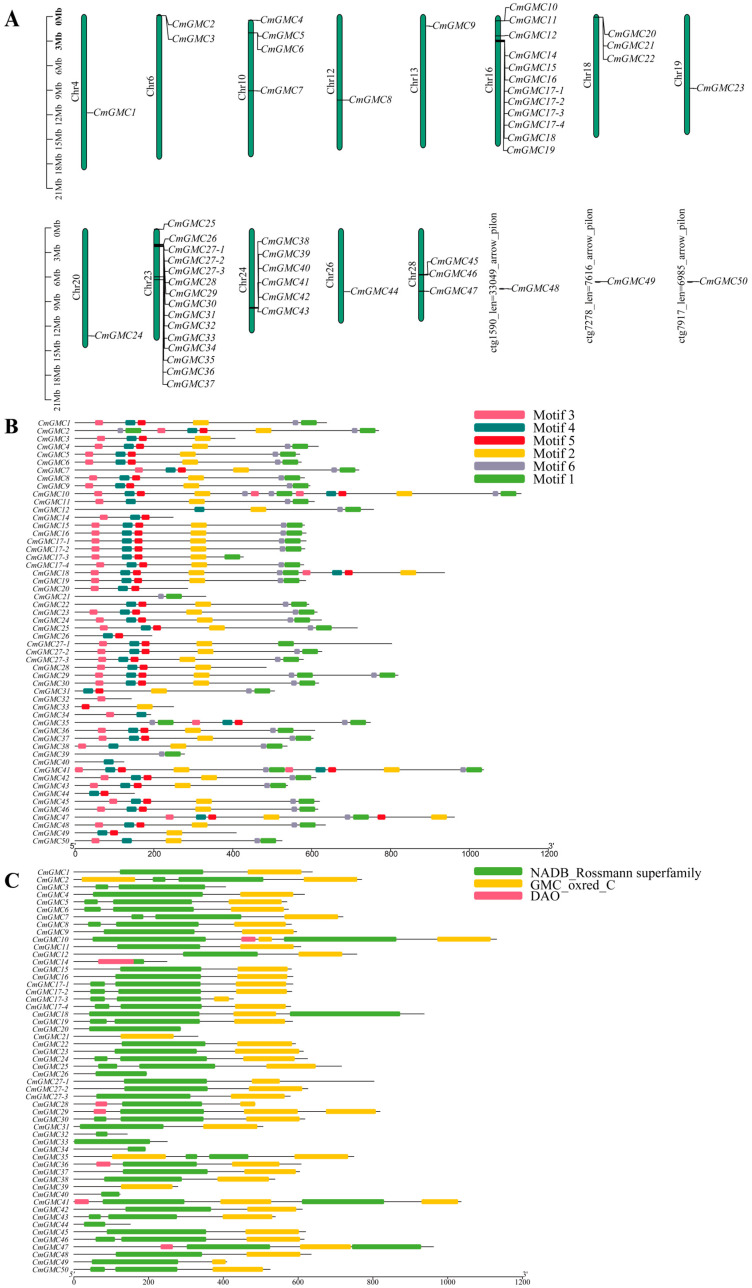
Bioinformatics analysis of the GMC gene family of *Cnaphalocrocis medinalis*. (**A**) Chromosome localization analysis, (**B**) motif analysis, and (**C**) conserved domain analysis were conducted on 54 GMC oxidoreductase family genes of *C. medinalis* using TBtools. Conserved motif analysis of the GMC gene family proteins. The figure displays six conserved motifs, with boxes of different colors representing distinct motifs and their respective positions and lengths within the protein sequences. Conserved domain analysis of the GMC gene family proteins. The green boxes represent the NADB_Rossmann superfamily domain, the yellow boxes represent the GMC_oxred_C domain, and the red boxes represent the DAO domain. The lengths of the domains are depicted proportionally to their occurrence in the protein sequences.

The 54 CmGMC genes were unevenly distributed across 13 chromosomes and three unplaced scaffolds. Three genes (*CmGMC48*, *CmGMC49*, *CmGMC50*) were each located on different unplaced scaffolds, while the remaining genes were mapped to assembled chromosomes. Multiple genes were located on chromosomes 6, 10, 16, 18, 23, 24, and 28 (Figure 2A); with the exception of chromosome 28, the other six chromosomes contained gene clusters. Chromosome 16 contained two gene clusters, while the other aforementioned chromosomes contained only one cluster each. The gene cluster on chromosome 23 contained 12 genes (*CmGMC26* to *CmGMC35*), representing the largest GMC gene cluster in the *C. medinalis* genome. Gene lengths ranged from 431 bp (*CmGMC32*) to 27,552 bp (*CmGMC47*) (Table A3). Four shorter genes (*CmGMC32*, *CmGMC33*, *CmGMC39*, *CmGMC44*) had lengths of less than 1 kb; such genes might encode small functional RNAs or short peptides or have important regulatory functions. Additionally, eight genes (*CmGMC10*, *CmGMC25*, *CmGMC29*, *CmGMC36*, *CmGMC37*, *CmGMC41*, *CmGMC45*, *CmGMC47*) were longer than 10 kb; these genes might be involved in complex functions, such as encoding large proteins that participate in various biological processes.

Similar motifs often indicate similar functions. Motif analysis identified six potentially functional motif modules (Figure 2B). Thirty-three genes contained all six motifs. Only four genes (*CmGMC21*, *CmGMC32*, *CmGMC33*, *CmGMC39*) lacked motif 4. Furthermore, motifs 2 and 6 were always found together, suggesting these two motifs might function cooperatively or even belong to the same functional module.

The GMC gene family typically contains two main conserved domains: the NADB_Rossmann superfamily and the GMC_oxred_C domain (Figure 2C), each containing an active site. In *C. medinalis*, 43 genes contained both of these conserved domains. *CmGMC21* and *CmGMC39* contained only the GMC_oxred_C domain, which is located at the C-terminus of the enzyme and is primarily involved in binding flavin adenine dinucleotide (FAD) and the oxidation of substrates. Nine genes (*CmGMC3*, *CmGMC14*, *CmGMC20*, *CmGMC26*, *CmGMC32*, *CmGMC33*, *CmGMC34*, *CmGMC40*, and *CmGMC44*) contained only the NADB_Rossmann superfamily domain, which contains the Rossmann fold, a highly conserved structure that binds NAD+, suggesting these genes might primarily participate in reduction reactions.

### 3.4. Phylogenetic Analysis of the C. medinalis GMC Oxidoreductase Family

**Figure 3:**
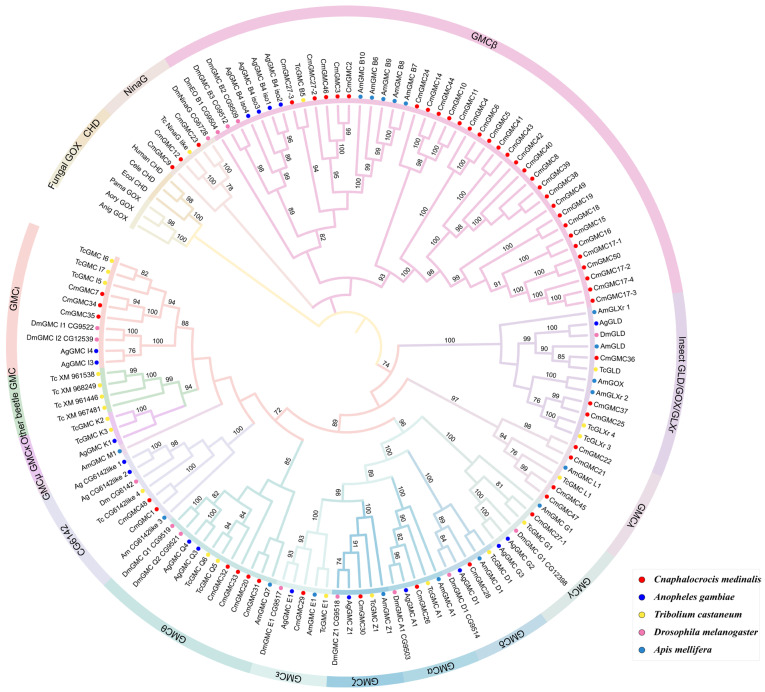
Phylogenetic analysis of the GMC gene family. The phylogenetic tree was constructed using the maximum likelihood method in MEGA12 software (v12.1.1) with the LG + G + I model across 1000 bootstrap replicates; nodes with less than 70% bootstrap support are not shown. Solid circles of different colors represent different species, while the different subfamilies are indicated by color-coded branches and arcs surrounding the tree.

A phylogenetic tree was constructed using GMC oxidoreductase genes from five species: *C. medinalis*, *D. melanogaster*, *T. castaneum*, *A. gambiae*, and *A. mellifera*. The tree comprised three major groups (Figure 3). The first group primarily consisted of genes from distantly related outgroups that included bacteria and mammals, indicating that insect GMC genes are very dissimilar from those in bacteria and fungi and thus may have an independent origin. The entire β subfamily and the NinaG subfamily formed the second group. The NinaG subfamily is the most closely related to the β subfamily among all 15 subfamilies; within the clustered genes in this group, three out of five genes were from *C. medinalis*, indicating the growth of this subfamily in Lepidoptera. Thirty of the 54 *C. medinalis* GMC genes were determined to be within the β subfamily. This group contained three smaller subgroups, one of which contained 24 *C. medinalis* genes, indicating that the expansion of the GMC family in *C. medinalis* primarily originated from the expansion of the β subfamily. The third group contained 13 subfamilies. Three of these subfamilies (GMCκ, Other beetle GMC, GMCμ) contained no *C. medinalis* GMC genes. The GMCκ subfamily contained two genes from *T. castaneum* and one from *A. gambiae*. The ‘Other beetle GMC’ subfamily consisted mainly of GMC genes from the coleopteran *T. castaneum*, and these two subfamilies were closely related phylogenetically, primarily comprising beetle GMC genes. This suggests that the functional differentiation of these two subfamilies might have occurred during the evolution of Coleoptera. The GMCμ subfamily was unique, containing only a single gene belonging to *A. mellifera*, suggesting this gene is functionally conserved within this lineage. The remaining ten subfamilies contained varying numbers of CmGMC genes (one to four each). The 12 genes from the conserved gene cluster (*CmGMC26* to *CmGMC35*) were distributed across nine of these subfamilies (excluding CG6142), indicating that this gene cluster underwent multiple duplication events and diversified into multiple genes that were shaped and maintained by natural selection in the lineage leading to *C. medinalis*.

### 3.5. Spatiotemporal Expression Analysis of the GMC Gene Family

**Figure 4:**
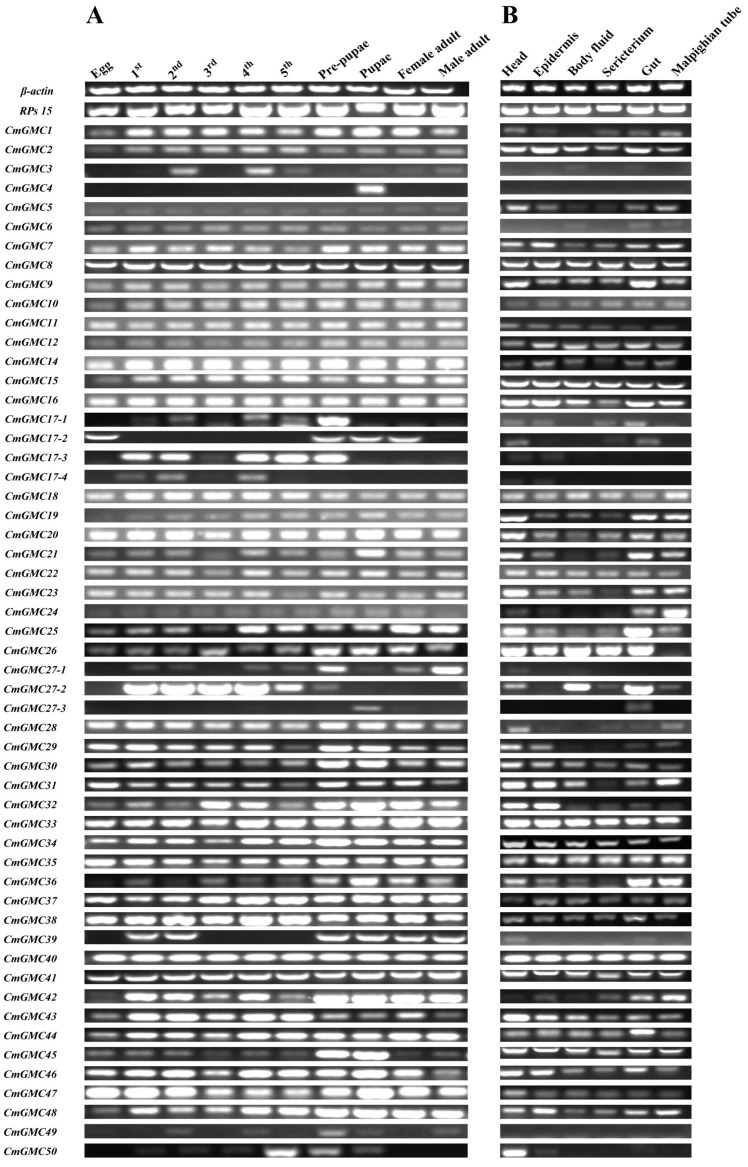
Spatiotemporal expression profiles of *Cnaphalocrocis medinalis* GMC genes. (**A**) Temporal expression profiles of CmGMC genes across different developmental stages; (**B**) Spatial expression profiles of CmGMC genes across different tissues.

As indicated by phylogenetic analysis, the expansion of the GMCβ subfamily in *C. medinalis* led to the observed increase in GMC members. PCR detection of the 54 GMC genes indicated that most genes are indeed transcribed (Figure 4). Expression analysis of GMC genes across different developmental stages (egg, first through fifth instar larvae, prepupa, pupa, and male and female adults) and tissues (head, epidermis, body fluid, sericterium, gut, Malpighian tubule) of *C. medinalis* revealed that 38 CmGMC oxidoreductase genes were expressed across all stages and tissues, indicating high overall transcriptional activity of CmGMC genes in *C. medinalis*. *CmGMC1* and *CmGMC28* were expressed in all tissues except hemolymph, while *CmGMC26* was expressed in all tissues except Malpighian tubules, suggesting these genes do not function in these respective tissues. *CmGMC4* was expressed only in pupae, indicating this gene primarily has pupae-specific functions. *CmGMC17-4* and *CmGMC49* were not expressed in any of the six assayed tissues. In the temporal expression profile, only 43 genes were expressed in eggs, while 51 genes were expressed in fourth instar larvae (except *CmGMC4*, *CmGMC39*, and *CmGMC27-3*). In the spatial expression profile, the greatest number of expressed genes (49) was observed in the head (except *CmGMC3*, *CmGMC4*, *CmGMC6*, *CmGMC17-4*, and *CmGMC49*); while hemolymph showed the lowest (42).

### 3.6. Response of the C. medinalis GMC Family Genes to Spinetoram

#### 3.6.1. Biological Determination of Spinetoram on *C. medinalis*

**Figure 5:**
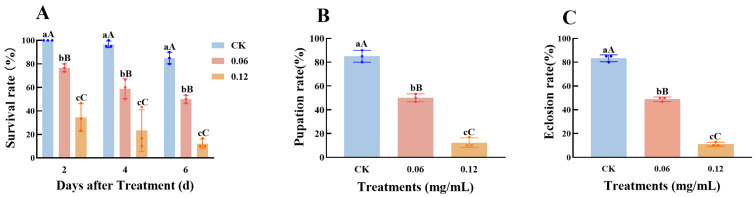
Bioassay of third-instar *Cnaphalocrocis medinalis* larvae treated with spinetoram. (**A**) Survival rate of third-instar larvae following spinetoram treatment. (**B**) Pupation and (**C**) eclosion rates of *C. medinalis* after spinetoram treatment. Data represent mean ± SE of *n* = 3 biological replicates, with 30 larvae per replicate. Different lowercase letters indicate significant differences among time points according to Tukey’s test (*p* ≤ 0.05); Different capital letters indicate significant differences among time points according to Tukey’s test (*p* < 0.01).

Third-instar *C. medinalis* larvae were treated with spinetoram at concentrations of 0.06 and 0.12 mg/mL. Survival rates were significantly lower in the two spinetoram-treated groups compared to the control at 2 d post treatment (Figure 5A) and decreased further by 4 and 6 d post treatment. Survival rates were significantly higher in the 0.06 mg/mL treatment group compared to the 0.12 mg/mL group. Concurrently, pupation (Figure 5B) and eclosion rates (Figure 5C) in the treatment groups were also significantly lower than in the control group, with the eclosion rate being only 11% after 0.12 mg/mL treatment, indicating the strong toxicity of spinetoram against *C. medinalis*. The two different concentrations of spinetoram imposed distinctly different levels of stress on *C. medinalis* survival, with the 0.12 mg/mL dose inducing a significantly stronger toxic response than the 0.06 mg/mL dose. To more accurately assess the role of the GMC gene family in the response to spinetoram, we selected the 0.06 mg/mL concentration for subsequent gene expression analysis.

#### 3.6.2. The Relative Expression of CmGMC Against to Spinetoram in *C. medinalis*

**Figure 6:**
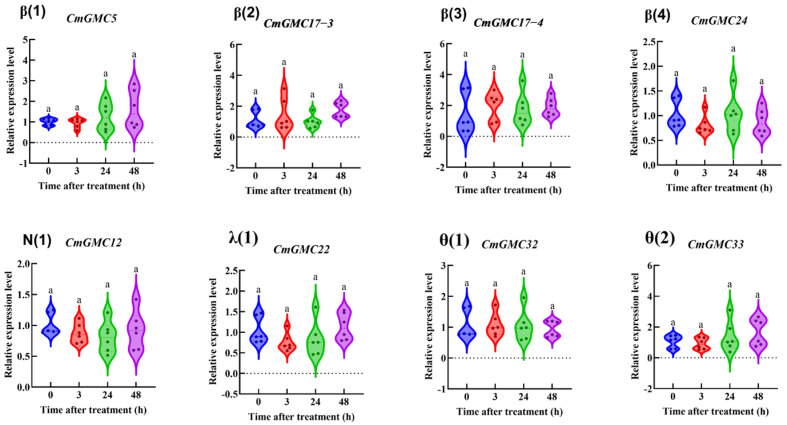
The relative expression level of genes at different time points after insecticide treatment. Data represent mean ± SE of *n* = 3 biological replicates, with 3 larvae per replicate. Each data point represents the mean value calculated from two internal reference genes. Bars with same letter means “a” no significant difference among different time points according to Tukey’s test (*p* ≤ 0.05).

As survival rates significantly decreased after spinetoram treatment, we measured the expression levels of all 54 CmGMC genes at 0, 3, 24, and 48 h post treatment (hpt) via qRT-PCR to identify genes that responded to spinetoram. Eight genes exhibited no significant difference in expression before and after treatment (Figure 6).

**Figure 7:**
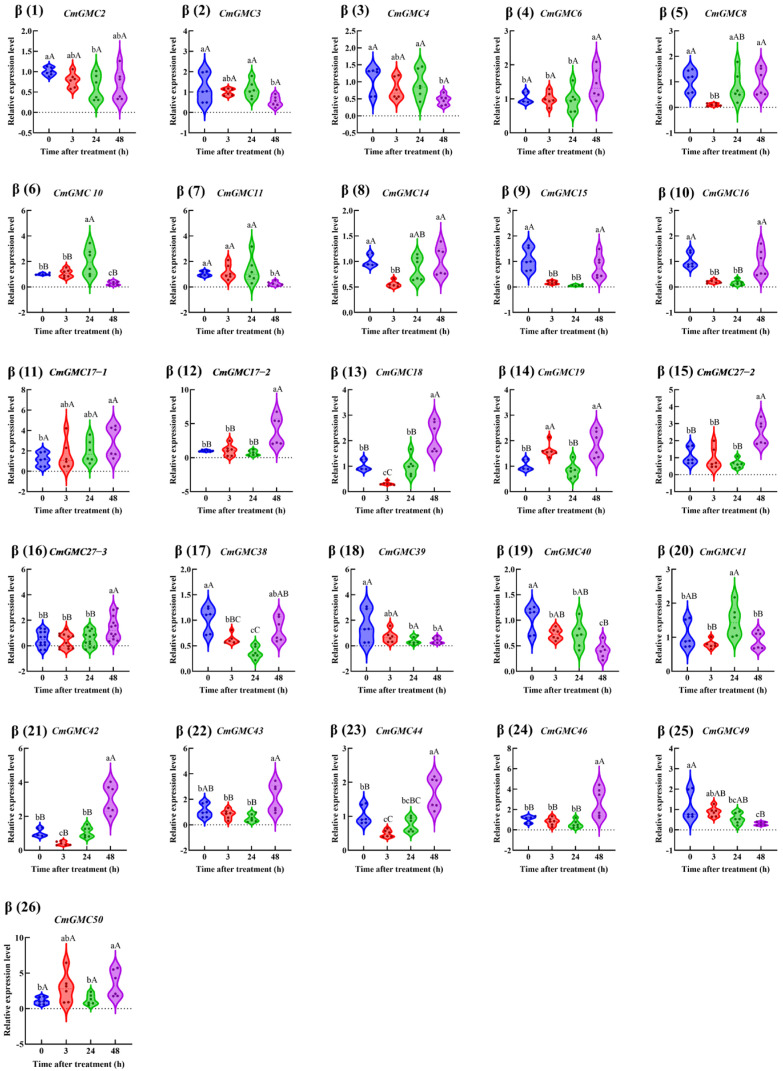
The relative expression level of the GMCβ subfamily genes at different time points after insecticide treatment. Data represent mean ± SE of *n* = 3 biological replicates, with 3 larvae per replicate. Each data point represents the mean value calculated from two internal reference genes. Different lowercase letters indicate significant differences among time points according to Tukey’s test (*p* < 0.05). Different capital letters indicate significant differences among time points according to Tukey’s test(*p* < 0.01).

The remaining 46 genes were categorized according to their phylogenetic subfamilies. Within the β subfamily (Figure 7), the expression of ten genes (*CmGMC8*, *CmGMC14*, *CmGMC15*, *CmGMC16*, *CmGMC18*, *CmGMC38*, *CmGMC40*, *CmGMC 42*, *CmGMC 44*, *CmGMC 49*) was suppressed at 3 hpt. At 24 hpt, *CmGMC10* and *CmGMC41* expression increased significantly, *CmGMC2*, *CmGMC15*, *CmGMC 16*, *CmGMC 38*, *CmGMC 39*, *CmGMC 40* expression decreased significantly. At 48 hpt, thirteen genes (*CmGMC 6*, *CmGMC 17-1*, *CmGMC17-2*, *CmGMC18*, *CmGMC19*, *CmGMC27-2*, *CmGMC27-3*, *CmGMC42*, *CmGMC44*, *CmGMC46*, *CmGMC49*, *CmGMC 50*) were actively transcribed, showing increased expression, while the expression levels of *CmGMC 3*, *CmGMC4*, *CmGMC 10*, *CmGMC 11*, *CmGMC 39* and *CmGMC40* decreased significantly. Thus, GMC gene expression was initially suppressed at 24 hpt after spinetoram treatment.

**Figure 8:**
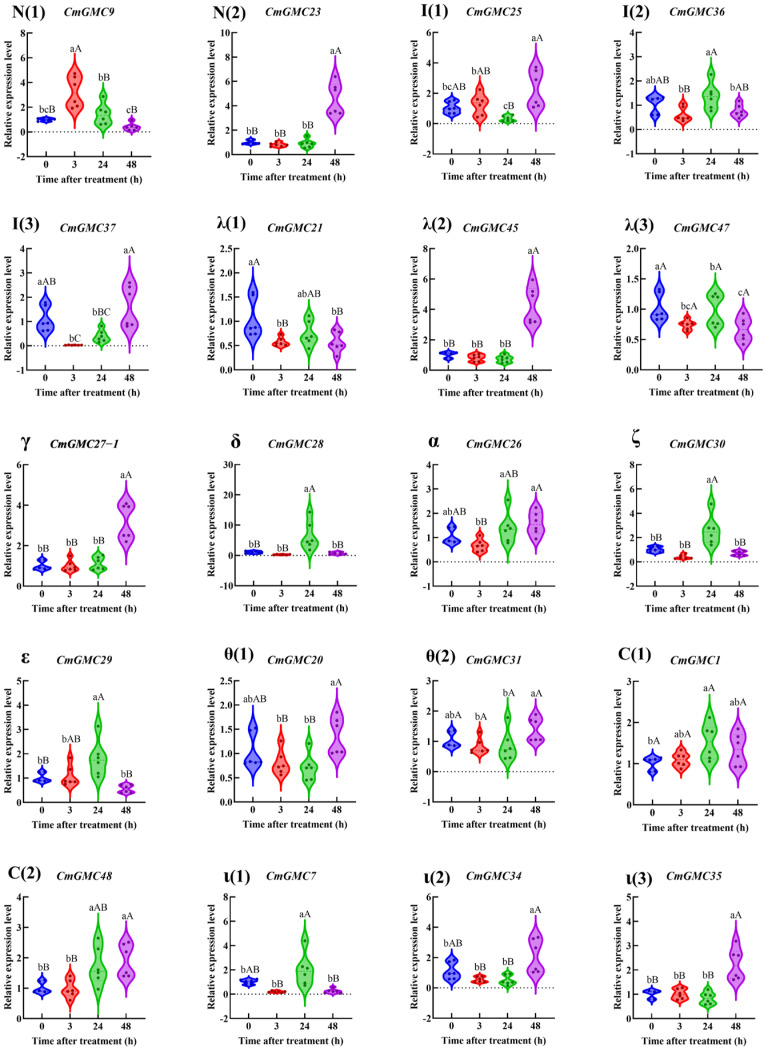
The relative expression level of GMC genes other than β subfamily genes at different time points after spinetoram treatment. Data represent mean ± SE of *n* = 3 biological replicates, with 3 larvae per replicate. Each data point represents the mean value calculated from two internal reference genes. Different lowercase letters indicate significant differences among time points according to Tukey’s test (*p* < 0.05). Different capital letters indicate significant differences among time points according to Tukey’s test (*p* < 0.01). Greek letters indicate the different subfamilies, with N (1–2) and I (1–3) denoting the NinaG and Insect GLD/GOX/GLXr subfamilies, respectively.

Within the remaining 11 subfamilies (Figure 8), a total of 20 genes showed altered expression. Among them, *CmGMC9* exhibited increased expression at 3 hpt, while the expression levels of both *CmGMC21*, *CmGMC37* and *CmGMC47* decreased. At 24 hpt, the expression levels of *CmGMC28*, *CmGMC29*, *CmGMC30*, *CmGMC1*, *CmGMC48* and *CmGMC7* increased significantly. At 48 hpt, the expression levels of 7 genes (*CmGMC23*, *CmGMC25*, *CmGMC45*, *CmGMC27-1*, *CmGMC48*, *CmGMC34* and *CmGMC35*) increased, suggesting that these 7 genes are primarily activated at 48 hpt when *C. medinalis* is affected by spinetoram toxicity, playing important roles after spinetoram enters the body.

## 4. Discussion

The rice leaf folder, *Cnaphalocrocis medinalis*, is one of the three major pests that currently reduces rice production in Asia. However, as a consequence of the overuse of chemical control agents, pesticide resistance in this species has reached high levels in some regions. Notably, pesticide exposure often induces oxidative stress. To this end, we identified 54 GMC oxidoreductase family genes in the *C. medinalis* genome. As observed in other lepidopteran species, the GMCβ subfamily in *C. medinalis* showed dramatic expansion relative to other insects. By constructing spatiotemporal expression profiles of the GMC gene family, we found that most GMC genes are transcriptionally active, with 38 genes expressed across all developmental stages and tissues examined. Analysis of *C. medinalis* treated with a sublethal concentration of spinetoram (LC_25_, 0.06 mg/mL) revealed that 8 genes showed no change in expression, while at 48 hpt, 20 and 8 genes were upregulated and downregulated, respectively. These genes may play a key role in the defense of *C. medinalis* against spinetoram toxicity [29].

The variation in physicochemical properties may underlie their functional differentiation. Throughout evolution, the homologous copies of a single ancestral gene that originally arose through the processes of both gene duplication and speciation can become altered through base mutations, insertions, and deletions, leading to functional divergence and novel functions, thereby giving rise to gene families [61]. The occurrence of acidic and basic proteins within a gene family, resulting from duplication and divergence, is indicative of functional diversity. This phenomenon occurs in many gene families. For example, in the histone superfamily, core histones are primarily basic, whereas HMG proteins are acidic; they evolved from a single-copy ancestral protein into proteins with nearly opposite charges, related to their divergent functions [62,63]. The instability index was less than 40 for the majority of the *C. medinalis* GMC proteins, indicating good overall stability of these proteins in *C. medinalis*. This abundance of unstable proteins is relatively uncommon among insect gene families. For example, 17 out of 31 HSPs in *Callosobruchus chinensis* had an instability index less than 40 [64], and 10 out of 13 FMO proteins in *Bursaphelenchus xylophilus* had indices below 40 [65].

The GMC gene family is a large family primarily involved in redox reactions, which are among the most crucial reactions in biological organisms. The heterogeneity in motifs among family members underscores the functional diversity of this family [66]. An aliphatic index below 60 indicates a low proportion of aliphatic amino acids, high hydrophilicity, and relatively poor thermal stability; 60 to 90 is the common range for most natural proteins; an index greater than 90 indicates high hydrophobicity and high thermal stability [67]. The aliphatic indices of the *C. medinalis* GMC family genes ranged from 61.3 to 105.1, indicating their high thermal stability.

The number of GMC genes in *C. medinalis* is relatively high among species in which GMC genes have been identified [28]. This gene family was first discovered in bacteria, with subsequent discoveries in fungi and plants [5]. In recent years, GMC genes have been gradually identified in insects, particularly within Lepidoptera, where the GMC family shows substantial expansion relative to other insects. In the silkworm, *B. mori*, the GMC family comprises 43 genes as a consequence of the expansion of the β subfamily [9]. A total of 257 GMC genes have been identified across five lepidopteran species: *S. litura*, *M. sexta*, *Pieris rapae*, *C. virescens*, and *D. plexippus* [30]. In both *D. melanogaster* and *B. mori*, multiple GMC genes have been observed to be tandemly arrayed within the introns of the *flotillin-2* gene [28,29]. We identified the *Flotillin-2* gene on chromosome 23 using the HMM, but the *C. medinalis* gene cluster was not found to be located within this gene. Whether this is due to unique genes in *C. medinalis* or other underlying mechanisms remains to be explored. Phylogenetic analysis revealed that the *C. medinalis* genes are distributed among 12 different subfamilies, with the majority belonging to the β subfamily, which contains 30 genes. The 54 GMC genes in *C. medinalis* are unevenly distributed across 13 chromosomes and three unplaced scaffolds, with most genes forming gene clusters, indicating a history of tandem gene duplication events, primarily within the β subfamily.

Thus, the expansion of the GMC gene family was mostly associated with tandem duplication events. A similar phenomenon was observed in *Parnassius glacialis*, in which the NPC2 gene family underwent significant expansion through tandem duplication [68]. Furthermore, this phenomenon is widespread among other gene families; expansions in the odorant receptor (Or) and gustatory receptor (Gr) gene families in *D. melanogaster* [69], the UTP-glucose-1-phosphate uridylyltransferase (UGPase) gene family in *Pseudoregma bambucicola* [70], and the Tret gene family in *B. mori* [71] have primarily been driven by tandem gene duplication events. Similarly, detoxification enzyme gene families like P450s, GSTs, and UDP-glucuronosyltransferases underwent significant expansion in *A. glabripennis*, which is related to their ability to degrade complex defensive compounds in wood [72]. Pit vipers and pythons acquired the ability to detect infrared radiation, enabled by the expansion of a specific TRPA1 ion channel subfamily [73]. Similarly, we hypothesize that the expansion of the β subfamily in Lepidoptera may underlie its role in the adaptation of Lepidoptera to its environment. In insects, β subfamily genes are associated with development [30], stress resistance [74], and immunity [29], among other functions.

Determination of the spatiotemporal expression profile of GMC genes in *C. medinalis* revealed that most GMC genes are expressed across multiple stages and tissues, with consistently high transcriptional activity. This indicates that GMC genes likely play indispensable roles in *C. medinalis*. Similarly, gene families like ribosomal proteins and cytoskeletal proteins are widely expressed across all tissues in humans [75], as are members of the P450 gene family [76] and heat shock protein (HSP) family in insects [77]; they are involved in various crucial functions, such as growth, development, and stress resistance.

To further investigate the response of GMC genes in *C. medinalis* to insecticide, we measured the relative expression levels of these genes at 3, 24, and 48 h after spinetoram treatment. We found that the expression levels of multiple genes changed, with 20 GMC genes upregulated. This finding is consistent with observations in *D. melanogaster* [78], the brown planthopper (*N. lugens*) [79], and the whitefly (*B. tabaci*) [80], in which detoxification enzyme gene expression has been reported as upregulated under pesticide stress. These enzymes can rapidly degrade pesticides into non-toxic or less toxic substances and facilitate their excretion, thereby mitigating pesticide stress. This is also an important molecular mechanism of insecticide resistance in insects. The three main classes of detoxification enzyme gene families in insects are P450, GSTs, and CarEs [81]. P450s primarily introduce an oxygen atom into a substrate molecule, thereby increasing its water solubility and thus facilitating either the subsequent action of detoxification enzymes to process them or direct excretion [82]. The core function of GSTs is catalyzing the conjugation of glutathione with various electrophilic, hydrophobic toxic compounds, thus promoting their water solubility and subsequent excretion [83]. CarEs mainly act in two ways: firstly, hydrolases break down insecticides into non-toxic or less toxic acids and alcohols; secondly, CarE proteins preferentially bind to insecticide molecules, thus preventing them from reaching their target substrates [84]. Currently, there are no definitive published studies that conclusively identify specific GMC oxidoreductase genes responsible for insecticide detoxification. However, a previous study has shown that Jinggangmycin-induced reproductive inhibition in the small brown planthopper, *L. striatellus*, is mediated by GDH via synthesis metabolism of fatty acid pathways [10]. Gut regurgitant bacteria (*Staphylococcus haemolyticus* and *Enterobacter*) in the diamondback moth, *P. xylostella*, inhibit development of the moth by suppressing the expression of glucose oxidase [85]. Similarly, another study found that suppressing the expression of the 20-hydroxyecdysone (20E) degradation enzyme gene, glucose dehydrogenase (PxGLD), in the gut of *P. xylostella* can enhance the resistance of this insect species to *Bt* insecticidal proteins [86]. These genes all belong to the GMC oxidoreductase family, indicating the important roles of this family in insect defense against chemical agents. In *C. medinalis*, 15 GMC genes were upregulated and 3 GMC genes were downregulated in response to spinetoram treatment. Thus, spinetoram likely activates or suppresses the expression of these genes, thereby regulating the growth and development of *C. medinalis* under the stress induced by this chemical control agent.

## 5. Conclusions

We identified a total of 54 GMC oxidoreductase family genes in *Cnaphalocrocis medinalis*, most of which were determined to be transcriptionally active. Among this family, the β subfamily contains 30 genes. Treatment with a sublethal dose of spinetoram altered the expression of 26 genes. Whether the expansion of the GMCβ gene contributes to detoxification and resistance remains to be validated, and the underlying physiological, biochemical, and molecular mechanisms of their action require further investigation.

## Figures and Tables

**Table 1:** Analysis of physical and chemical properties of the GMC Genes of *Cnaphalocrocis medinalis*.

Gene ID	Gene Name	AA	MW	PI	II	AI	GRAVY
*Cmed12162*	*CmGMC1*	638	70,952.5	9.2	31.0	84.3	−0.198
*Cmed14810*	*CmGMC2*	770	85,725.5	5.3	28.6	92.2	−0.178
*Cmed14811*	*CmGMC3*	406	44,942.7	5.0	33.3	90.8	−0.136
*Cmed12844*	*CmGMC4*	617	69,151.7	8.3	32.7	97.2	−0.092
*Cmed12391*	*CmGMC5*	570	63,769.6	8.6	38.6	80.9	−0.385
*Cmed12392*	*CmGMC6*	574	63,690.0	5.9	44.1	84.9	−0.315
*Cmed07297*	*CmGMC7*	720	80,319.7	6.8	41.2	83.0	−0.373
*Cmed04604*	*CmGMC8*	582	63,256.7	5.8	39.0	90.8	−0.096
*Cmed21934*	*CmGMC9*	596	65,188.4	6.1	23.6	92.0	−0.071
*Cmed03671*	*CmGMC10*	1131	127,085.0	8.8	36.5	85.7	−0.368
*Cmed03670*	*CmGMC11*	607	68,535.8	9.3	33.9	85.8	−0.401
*Cmed13421*	*CmGMC12*	757	85,214.7	6.2	43.3	90.0	−0.134
*Cmed16067*	*CmGMC14*	249	27,478.8	4.6	16.9	77.2	−0.325
*Cmed17247*	*CmGMC15*	582	64,279.7	5.1	33.9	85.1	−0.274
*Cmed17244*	*CmGMC16*	586	64,749.0	8.9	26.6	86.8	−0.250
*Cmed15861.1*	*CmGMC17−1*	585	65,046.3	9.0	34.4	87.3	−0.280
*Cmed15861.2*	*CmGMC17−2*	582	64,833.4	5.9	35.7	82.6	−0.304
*Cmed15861.3*	*CmGMC17−3*	426	47,104.8	9.1	35.9	87.9	−0.256
*Cmed15861.4*	*CmGMC17-4*	579	64,737.8	8.8	44.7	82.2	−0.266
*Cmed15863*	*CmGMC18*	937	104,026.8	8.9	35.5	87.9	−0.319
*Cmed15865*	*CmGMC19*	585	65,085.5	7.0	34.5	91.0	−0.192
*Cmed00507*	*CmGMC20*	286	32,065.3	6.2	27.3	76.6	−0.395
*Cmed00506*	*CmGMC21*	332	36,709.6	8.6	37.4	105.1	−0.031
*Cmed00509*	*CmGMC22*	593	66,415.3	6.2	29.8	88.3	−0.191
*Cmed07035*	*CmGMC23*	614	68,822.4	9.6	42.9	84.5	−0.219
*Cmed01344*	*CmGMC24*	625	69,630.7	8.5	41.6	89.3	−0.224
*Cmed05264*	*CmGMC25*	716	79,697.7	5.7	42.1	68.1	−0.452
*Cmed14944*	*CmGMC26*	195	21,903.8	6.7	32.3	73.5	−0.492
*Cmed14940.1*	*CmGMC27−1*	802	90,806.6	5.6	37.3	80.3	−0.449
*Cmed14940.2*	*CmGMC27−2*	625	69,200.3	8.9	27.6	81.0	−0.320
*Cmed14940.3*	*CmGMC27−3*	578	64,439.1	5.8	35.2	94.5	−0.156
*Cmed14941*	*CmGMC28*	485	54,007.8	8.4	39.6	88.7	−0.188
*Cmed14939*	*CmGMC29*	819	91,141.9	8.8	37.5	85.6	−0.209
*Cmed07555*	*CmGMC30*	618	69,003.5	9.3	33.1	91.0	−0.183
*Cmed07559*	*CmGMC31*	506	56,640.1	9.3	32.3	79.8	−0.347
*Cmed07562*	*CmGMC32*	143	15,617.8	6.1	36.7	90.7	−0.068
*Cmed07560*	*CmGMC33*	250	28,430.6	9.7	33.1	79.5	−0.457
*Cmed07563*	*CmGMC34*	192	21,392.6	9.1	40.3	74.2	−0.398
*Cmed07558*	*CmGMC35*	749	82,434.1	5.1	33.7	81.3	−0.250
*Cmed00473*	*CmGMC36*	608	65,919.9	6.9	40.1	71.6	−0.274
*Cmed14455*	*CmGMC37*	604	67,148.4	7.2	35.9	76.4	−0.397
*Cmed06630*	*CmGMC38*	538	59,350.0	5.2	44.0	86.8	−0.283
*Cmed06631*	*CmGMC39*	278	30,434.6	5.7	50.3	89.1	−0.072
*Cmed06632*	*CmGMC40*	124	13,947.8	5.2	43.8	86.5	−0.173
*Cmed06636*	*CmGMC41*	1036	113,781.1	5.8	35.1	85.8	−0.220
*Cmed06637*	*CmGMC42*	611	66,566.3	6.8	40.4	93.4	−0.101
*Cmed06633*	*CmGMC43*	539	59,604.8	6.2	39.0	87.7	−0.176
*Cmed16057*	*CmGMC44*	151	17,369.3	5.8	10.0	61.3	−0.837
*Cmed22270*	*CmGMC45*	620	69,341.7	8.2	28.2	81.6	−0.394
*Cmed22269*	*CmGMC46*	616	68,593.7	5.3	34.6	87.7	−0.173
*Cmed06807*	*CmGMC47*	962	108,670.7	9.2	40.7	74.4	−0.443
*Cmed04144*	*CmGMC48*	635	70,851.6	9.5	32.5	84.1	−0.224
*Cmed18776*	*CmGMC49*	409	45,325.4	4.9	39.1	77.5	−0.357
*Cmed19817*	*CmGMC50*	525	58,163.6	9.1	37.1	84.9	−0.310

Note: AA, number of amino acids encoded by the gene; MW, relative molecular weight of the encoded protein; PI, theoretical isoelectric point; II, instability index; AI, aliphatic index, indicating thermal stability; GRAVY, grand average of hydrophobicity.

## Data Availability

Data is contained within the article and supporting file. The original data can be obtained by contacting the author via 13213502940@163.com.

## Supplementary Materials

The following supporting information can be downloaded at: https://www.mdpi.com/article/10.3390/insects16121272/s1.

## Abbreviations

The following abbreviations are used in this manuscript:

GMC	Glucose–Methanol–Choline
CmGMC	Glucose–Methanol–Choline of *Cnaphalocrocis medinalis*.
LC50	Lethal Concentration 50
hpt	hours post treatment

## Appendix A

### Appendix A.1. Primers Used in the Study

The primers used in the article are all listed in Table A1. The primers were all designed in the Primer5 software.

**Table A1:** Primers used in PCR and qRT-PCR.

Gene	Forward Primer (5′→3′)	Reverse Primer (5′→3′)
*β-actin*	ATGGTCGGCATGGGACAG	GAGTTCATTGTAGAAGGTGT
*RPs15*	ACGTACCCGCTTACAAACCC	TGACCAAGGTGAGCAACAGAG
*CmGMC1*	GGAAGGGGCGGATACCTAGA	CCGTTCGGGTCTCTCCATTT
*CmGMC2*	ATCGGTCTTCCATCCATTGAC	AAAAAACCACGAGCTGTGCTC
*CmGMC3*	CAGGCCCCTCGTTTGACTTC	TAGGGTCGTCACCAGCTTCT
*CmGMC4*	CAGGTGCCTTTGCGATTGTT	TTGCTTCCACTCGATCCACC
*CmGMC5*	TATCGCCCCAATCTGCATG	TTTCCCGTTTGACAAAGCC
*CmGMC6*	GAAGAATCCAACATTCTCCCCATTG	TATGTTGAACAGTCTTTGATCGTCG
*CmGMC7*	GGAATCCCAGTCATTGCCGA	GGGCTCAAGTAAGCTGGCAT
*CmGMC8*	TGCCAGTGCCATGCCTAAT	TGCGCCAGTGTCCTTTTTAA
*CmGMC9*	TGCCAGTGCCATGCCTAAT	ATCAAAGTCCCGAGGGTTGC
*CmGMC10*	GTCTGCCACTCGCAGTTTGT	GGGACTGCTAGAATGGGTGAT
*CmGMC11*	GCTATCAATGGACGCGTTGG	GGTGGGCAGGCAAAGACATA
*CmGMC12*	CTGGCATTTATTGGAGCCTCA	GCCCCCACTTTCACTATCTTG
*CmGMC14*	CTTCATCGTTGTCGGAGCTG	CTATTGATTCCAAGGGTGGGT
*CmGMC15*	GGTTGCAGATGGAATGCGTC	CTTTGTCGCCGTTGTGTGTT
*CmGMC16*	TATGTGAGTACGTGTCGGATGG	GGTGTTACCGCTGGTGATGG
*CmGMC17-1*	ACAACCACCTGCACATGACT	TCCCCAGTCATATCAGCCCA
*CmGMC17-2*	TGCCTGGAACTACACCAACG	GCTCTTGTGACACCGACGTA
*CmGMC17-3*	CGTTCGAGGAGACCCTTACG	GGATCTTCCAGCCTTTCGCT
*CmGMC17-4*	ATGGCCTTCGTCAAGGTAGC	AGCTTGGACTGTGATGGTGT
*CmGMC18*	AAACACCACCAACCCCCAT	CGACAAATCTTTAGCAAACGACAC
*CmGMC19*	TTATGGAAGCCGTAGCTCGC	GGCATCTCCAATACTCCGCA
*CmGMC20*	CGTCCCTACGAAAGACTACCG	CTCCCAACACCTTCCCAGAT
*CmGMC21*	ATGAGCGATGTTGCTCCAGT	GGCGCACTAACGCTTTCATA
*CmGMC22*	CTGAACGGAAACGATGGTTG	AAGGTCCCTGCGTGGAATG
*CmGMC23*	TCGTATGTGTTCGCCGTTG	ATATCTCTTTTCCGCTTTCCCT
*CmGMC24*	GATCGAGTCGGGGTTCCTT	CGCGCACACGCTTATAATAA
*CmGMC25*	ATGCCCACAGTCGTTACTGG	ACTTCGGGACGGTTCTTCAC
*CmGMC26*	TGCTGTTGGAAGCTGGTGG	CCTTGGGGCTCAGTCTTGTACT
*CmGMC27-1*	ACAGCATGATGGAAGCGGAA	CAGCCCATTAGCTCGTTTGC
*CmGMC27-2*	CAACGCAAGCGGTCATCAAA	TAGTAGGGTTCCCTCCAGCC
*CmGMC27-3*	CTGGGCAGCTATTCGCAAAC	CCCGACCACGATGAAGTCAA
*CmGMC28*	GGGGCTATAGGATCCCCACA	TGGAACACAAGCCCTCCAAT
*CmGMC29*	GGCCAAGACACCGTATCACA	TCGCGCCGTTTATATCACGA
*CmGMC30*	ACGGGCAGATCGTGAGAAA	GGACGCAACAATAAAGGCA
*CmGMC31*	CCATTACAAGTACGATATACCACCA	ACTCTCAAACCCTTCACGCC
*CmGMC32*	CAGAACACCGACCCCATCTC	TCGTACTCGGTGAGGAGGTT
*CmGMC33*	CATGCTCCTTTCAGAACAACGA	AACGATACGCCTGACGGAAA
*CmGMC34*	AAGGCCAGCTGTATTCCGTC	CAGAGGACCCCAACGTAACC
*CmGMC35*	ATGGAAAACGAGCGTTGCT	CGATCCTATCCCAGTCTTGAGG
*CmGMC36*	GCCCTACTTCCTCAAGTCCG	TAGCTCAAAGGCGGGTGGTA
*CmGMC37*	AACTACCAACCCAAAGAGACAAAAG	CGACCATGAACCCAACAGGA
*CmGMC38*	TATGCAGCCGTATTCAACTGG	AGCGAGCGAAGTCTTTAAGGTA
*CmGMC39*	GCAAAAACCTCCACGACCAC	GGCACTGGGAATAGGTGAGG
*CmGMC40*	ATCACGGGACTCCAGCTCA	TCCATCGTTTTCGGAGGTGA
*CmGMC41*	CTGTGCTTCCAGGTCTGTTTCC	GCTGTCATTCCAGGCTTGTTC
*CmGMC42*	CTGAAACCACTTATTGAACGC	AATCCAAACTCCAACACCTT
*CmGMC43*	GTTGGAATCAGTGCTGCCT	TCCTTGAACGTTCTGTGCG
*CmGMC44*	GCAGATAAATTAACGCACGCTT	TAACTCGTCTGCCTTTGTGC
*CmGMC45*	GTATGGTGTTCACGGGCTCA	AAAACGAACGCCACTCCTCT
*CmGMC46*	TGGCCACGTGGAAAAACATT	CCCCAACCAAGGTTACCGAG
*CmGMC47*	GCAATTCCACAGTTACGGGC	TGCCAGGTTTGTATCCTTCGT
*CmGMC48*	CATGGTGGAAGGAGTACGATTAG	GGAAAGGGCACTGTGTGGA
*CmGMC49*	ATGGACTGGAACTACACGGC	CTGGTCGGTCTTGTGTCCTC
*CmGMC50*	TACCGGTATCCTTTGCCAGC	AGGGTCACCACCAGCTTCTA

### Appendix A.2. The LC50 Value of Spinetoram Against Cnaphalocrocis medinalis

The median lethal concentration (LC_50_) at 48 h and its 95% confidence limits for *C. medinalis* were determined using probit regression analysis. All statistical analyses were performed with the DPS statistical software (v18.10). The LC_50_ of spinetoram against third-instar larvae of *C. medinalis* at 48 h was 0.109 mg/L (95% CL: 0.088—0.133 mg/L). The goodness-of-fit test for the model indicated no significant deviation between the data and the model (χ^2^ = 1.594, df = 3, *p* = 0.661).

**Table A2:** The LC_50_ value of spinetoram against *Cnaphalocrocis medinalis*.

Pesticide	Y = a + bx	Slope ± SE	X^2^ (df)	R^2^	LC50 (95%CL) (mg/mL)
spinetoram	y = 1.78 + 1.84x	1.78 ± 0.201	1.594 (3)	0.987	0.109 (0.88–0.133)

Note: y represents the probability unit, and x is the logarithm (log10) of the concentration.

### Appendix A.3. Location of CmGMC in Cnaphalocrocis medinalis

The chromosomal positions, start sites, stop sites, lengths, and strand orientations of all GMC gene family members were extracted from the *C. medinalis* genome file using the “GXF Gene Position & Info Extract” tool in TBtools.

**Table A3:** Location of CmGMC in *Cnaphalocrocis medinalis*.

Sequence ID	Chr	Start	End	Length	±Chain
*CmGMC1*	Chr4	12,031,548	12,037,042	5494	−
*CmGMC2*	Chr6	153,967	158,617	4650	−
*CmGMC3*	Chr6	158,719	160,568	1849	−
*CmGMC4*	Chr10	55,831	58,252	2421	+
*CmGMC5*	Chr10	1,592,434	1,601,435	9001	−
*CmGMC6*	Chr10	1,601,490	1,605,747	4257	−
*CmGMC7*	Chr10	8,676,573	8,682,633	6060	+
*CmGMC8*	Chr12	10,471,975	10,474,609	2634	−
*CmGMC9*	Chr13	1,419,404	1,422,747	3343	+
*CmGMC10*	Chr16	632,936	643,480	10,544	+
*CmGMC11*	Chr16	645,155	647,221	2066	−
*CmGMC12*	Chr16	2,478,010	2,483,491	5481	−
*CmGMC14*	Chr16	2,993,030	2,997,453	4423	+
*CmGMC15*	Chr16	3,081,455	3,084,711	3256	+
*CmGMC16*	Chr16	3,093,006	3,097,729	4723	−
*CmGMC17−1*	Chr16	3,141,854	3,145,582	3728	−
*CmGMC17−2*	Chr16	3,147,393	3,149,605	2212	−
*CmGMC17−3*	Chr16	3,151,400	3,154,375	2975	−
*CmGMC17−4*	Chr16	3,155,559	3,158,119	2560	−
*CmGMC18*	Chr16	3,187,948	3,194,577	6629	−
*CmGMC19*	Chr16	3,201,984	3,208,334	6350	+
*CmGMC20*	Chr18	337,806	346,801	8995	+
*CmGMC21*	Chr18	346,848	347,911	1063	+
*CmGMC22*	Chr18	347,773	350,514	2741	−
*CmGMC23*	Chr19	9,029,185	9,036,379	7194	+
*CmGMC24*	Chr20	13,117,124	13,120,747	3623	−
*CmGMC25*	Chr23	169,852	183,234	13,382	+
*CmGMC26*	Chr23	2,013,736	2,017,128	3392	+
*CmGMC27−1*	Chr23	2,021,147	2,024,758	3611	+
*CmGMC27−2*	Chr23	2,027,013	2,030,765	3752	+
*CmGMC27−3*	Chr23	2,032,287	2,035,200	2913	+
*CmGMC28*	Chr23	2,057,813	2,063,102	5289	+
*CmGMC29*	Chr23	2,109,580	2,121,828	12,248	+
*CmGMC30*	Chr23	2,218,730	2,221,531	2801	+
*CmGMC31*	Chr23	2,246,861	2,251,496	4635	+
*CmGMC32*	Chr23	2,276,183	2,276,614	431	+
*CmGMC33*	Chr23	2,276,628	2,277,380	752	+
*CmGMC34*	Chr23	2,287,382	2,294,626	7244	+
*CmGMC35*	Chr23	2,296,315	2,304,712	8397	+
*CmGMC36*	Chr23	5,982,685	5,992,924	10,239	−
*CmGMC37*	Chr23	6,339,412	6,349,616	10,204	+
*CmGMC38*	Chr24	9,639,738	9,643,575	3837	−
*CmGMC39*	Chr24	9,654,959	9,655,795	836	−
*CmGMC40*	Chr24	9,656,209	9,657,796	1587	−
*CmGMC41*	Chr24	9,709,590	9,720,736	11,146	−
*CmGMC42*	Chr24	9,743,549	9,747,716	4167	−
*CmGMC43*	Chr24	9,750,697	9,753,458	2761	+
*CmGMC44*	Chr26	7,721,527	7,722,002	475	+
*CmGMC45*	Chr28	5,613,898	5,633,044	19,146	−
*CmGMC46*	Chr28	5,679,905	5,681,963	2058	+
*CmGMC47*	Chr28	7,652,152	7,679,704	27,552	−
*CmGMC48*	ctg1590	27,242	32,075	4833	+
*CmGMC49*	ctg7278	862	2515	1653	+
*CmGMC50*	ctg7917	15	4822	4807	+
